# READINESS FOR AGING: A CONCEPT ANALYSIS

**DOI:** 10.1093/geroni/igae098.3278

**Published:** 2024-12-31

**Authors:** Yanjing Liang, Basia Belza

**Affiliations:** University of Washington, Seattle, Washington, United States; University of Washington, Seattle, Washington, United States

## Abstract

As awareness of aging grows alongside healthcare advancements, advocacy for older adults’ quality of life intensifies. However, people focus more on the physiological, psychological, and social challenges associated with the aging process, limited attention has been paid to entering the pre-aging stage. Understanding of the phenomenon and concept of readiness for aging will enable us to appreciate the opportunities and challenges individuals face as they approach aging. This concept analysis conceptualizes readiness for aging. PubMed, Web of Science, PsycINFO, and CINAHL were systematically searched by using combinations of the terms “ready”, “readiness”, “prepared”, “prepare”, “preparedness”, “preparation”, “aging” and “ageing” in the abstract or title (from the earliest date available to February 2024). Rodgers’ approach was employed to clarify the antecedents, attributes, and consequences of readiness for aging. Fifteen eligible articles were reviewed. The findings were categorized into antecedents, attributes, and consequences. Antecedents of readiness for aging were classified into five categories: self-efficacy, social support, aging in place, financial preparedness, and health awareness. Attributes were characterized by psychological resilience, social connectedness, future orientation, financial plan, and health maintenance. The consequences of readiness for aging include quality of life, social well-being, financial security, and healthy aging. Given the limited attention readiness for aging has received, this concept analysis offered a refined and clarified conceptual definition of readiness for aging. Through literature reviews, qualitative and quantitative data collection, and collaboration with healthcare professionals, and legislators, future research can prioritize and enhance conceptual development, fostering holistic approaches to aging.

